# *De Novo* Sequencing of *Hypericum perforatum* Transcriptome to Identify Potential Genes Involved in the Biosynthesis of Active Metabolites

**DOI:** 10.1371/journal.pone.0042081

**Published:** 2012-07-30

**Authors:** Miao He, Ying Wang, Wenping Hua, Yuan Zhang, Zhezhi Wang

**Affiliations:** 1 Key Laboratory of the Ministry of Education for Medicinal Resources and Natural Pharmaceutical Chemistry, National Engineering Laboratory for Resource Development of Endangered Crude Drugs in Northwest of China, College of Life Sciences, Shaanxi Normal University, Xi’an, Shaanxi, People’s Republic of China; 2 Key Laboratory of Resource Biology and Biotechnology in Western China (Ministry of Education), Institute of Life Science, Northwest University, Xi’an, Shaanxi, People’s Republic of China; 3 Department of Life Sciences, Shaanxi Institute of Education, Xi’an, Shaanxi, People’s Republic of China; University of Hong Kong, China

## Abstract

**Background:**

*Hypericum perforatum* L. (St. John’s wort) is a medicinal plant with pharmacological properties that are antidepressant, anti-inflammatory, antiviral, anti-cancer, and antibacterial. Its major active metabolites are hypericins, hyperforins, and melatonin. However, little genetic information is available for this species, especially that concerning the biosynthetic pathways for active ingredients.

**Methodology/Principal Findings:**

Using *de novo* transcriptome analysis, we obtained 59,184 unigenes covering the entire life cycle of these plants. In all, 40,813 unigenes (68.86%) were annotated and 2,359 were assigned to secondary metabolic pathways. Among them, 260 unigenes are involved in the production of hypericin, hyperforin, and melatonin. Another 2,291 unigenes are classified as potential Type III polyketide synthase. Our BlastX search against the AGRIS database reveals 1,772 unigenes that are homologous to 47 known *Arabidopsis* transcription factor families. Further analysis shows that 10.61% (6,277) of these unigenes contain 7,643 SSRs.

**Conclusion:**

We have identified a set of putative genes involved in several secondary metabolism pathways, especially those related to the synthesis of its active ingredients. Our results will serve as an important platform for public information about gene expression, genomics, and functional genomics in *H. perforatum*.

## Introduction

*Hypericum perforatum* L. (common St. John’s wort) is a widely known medicinal herb used mostly as a remedy for depression [1]. It also has other broad pharmacological activities, such as anti-tumor, anti-inflammatory, antiviral, antioxidant, anti-cancer, and antibacterial properties [2], [3]. Human health is benefited because of this diversity of active ingredients within various chemical groups. Its major active metabolites – hypericins, hyperforins, and melatonin – belong to the naphthodianthrones, phloroglucinols, and alkaloids, respectively. Xanthones and flavonoids have also been identified in extracts from this plant [4].

*H. perforatum* has significant amounts of hypericin and hyperforin, which are considered to be most promising naturally occurring agents because of their important biological properties. Hypericins are the characteristic compounds of the genus *Hypericum* (Hypericaceae). Hyperforin has been found in significant amounts only in *H. perforatum* [5], whereas other *Hypericum* species contain only low levels of that compound [6]. Consequently, *H. perforatum* fascinates the researchers, and reveals huge market demand. Although the biosynthesis pathway leading to hypericins and hyperforins is still poorly understood, it is presumed that the type III polyketide synthase (PKS) is involved [7], [8]. This PKS family of enzyme complexes produces various polyketides in plants, including naphthodianthrones, phloroglucinols, xanthones, and flavonoids [4], [7], [8]. Type III PKSs catalyze the condensation between specific CoAs, such as acetyl-CoA and malonyl-CoA [9]. Based on their mechanisms of cyclization, these PKSs in higher plants are classified into three groups: chalcone synthase (CHS-type), stilbene synthase (STS-type), and coumaroyltriacetic acid synthase (CTAS-type) [9]. All have diverse functions that vary according to substrate preference, the amount of condensed malonyl-CoA, and the mechanism of cyclization reactions [10], [11].

**Figure 1:**
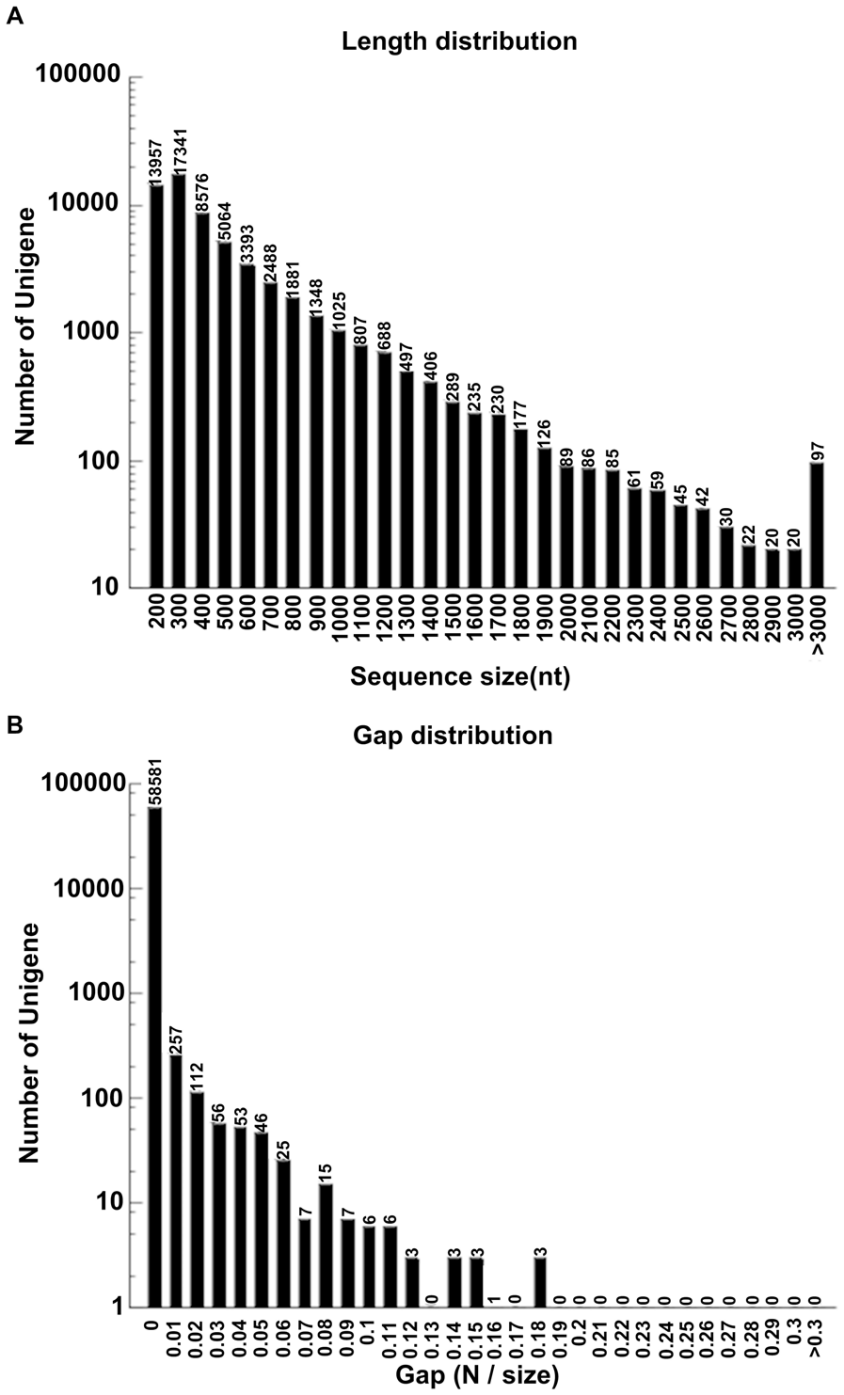
Distributions of lengths (**A**) **and gaps** (**B**) **for unigenes from** ***Hypericum perforatum*** **.**

Melatonin (N-acetyl-5-methoxytryptamine), a hormone secreted by the pineal gland in animal brains, helps regulate other hormones and maintain the body’s circadian rhythm [12]. It is also present in the plant kingdom [13], where it is considered an antioxidant or growth promoter [14]. Although its biosynthetic pathway is poorly understood, it is thought to be derived from tryptophan and serotonin [15]. Much current research has been focused on the detection, function, and biosynthesis of melatonin in *H. perforatum* because those plants produce significantly larger amounts of that hormone compared with other species [13].

Previous studies on *H. perforatum* have mainly involved its active ingredients and their pharmacological activities. Although much effort has been devoted to cloning and identifying the key enzymes for secondary metabolism in that species [16]–[19], only limited genomic information has been submitted to the National Center for Biotechnology Information (NCBI), i.e., 70 nucleotide sequences and 3 ESTs. Only a few of its genes function in secondary metabolism, and most studies have concentrated primarily on the Hyp-1 enzyme, which catalyzes hypericin biosynthesis. This is because traditional methods for gene cloning and sequencing are time-consuming, expensive, and produce only a little genetic information.

**Table 1:** Summary statistics of the sequence assembly generated from *Hypericum perforatum*.

Total number of reads	24,429,306
Total nucleotides (nt)	2,198,637,540
GC percentage	50.45%
Q20 percentage	94.62%
**Step-wise assembly**	
Total number of contigs	192,465
Average sequence size of contigs (bp)	204
Total number of scaffolds	115,587
Average sequence size of scaffolds (bp)	298
Total number of unigenes	59,184
Total nucleotides (nt) in unigenes	24,986,432
Average sequence size of unigenes (bp)	422

By contrast, RNA-Seq is a recently developed approach for profiling transcriptomes. It has many advantages because it is cost-effective, highly sensitive, more accurate, and has a large dynamic range [20]. It is now widely used to analyze gene expression and discover novel transcripts, SNPs, splice junctions, and fusion transcripts [21]–[23]. Here, we describe the utilization of Illumina/Solexa paired-end technology for *de novo* transcriptome analysis of *H. perforatum* throughout its life cycle. We obtained 2.2 GB of nucleotides and discovered almost all of the known genes for hypericin, hyperforin, and melatonin biosynthesis. The work presented here is the first to profile the genetic information of *H. perforatum*. Then it also provides an insight into the secondary metabolic pathways in that species, our results could be used for further genetic manipulation to improve its yield of active metabolites.

**Table 2:** Summary statistics of functional annotation for *Hypericum perforatum* unigenes in public protein databases.

Public protein database	Number of unigene hits	Percentage (%)
**NR**	40,551	68.52
**Swiss-Prot**	26,657	45.04
**COG**	11,209	18.94
**KEGG**	20,548	34.72
**Total**	40,813	68.86

**Figure 2:**
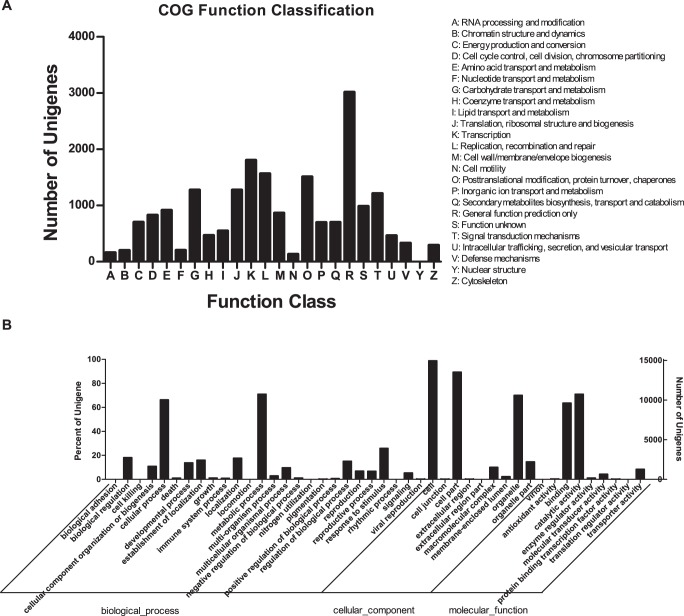
COG and GO classifications of unigenes derived via Solexa sequencing in *Hypericum perforatum*. (A), COG Function Classification of transcriptome. A total of 11,209 unigenes showing significant homology to COGs database at NCBI (E-value ≤1.0e^−5^) had COG classification among 24 categories. (B), *H. perforatum* unigenes with GO annotations based on *Arabidopsis* protein hits from NR. Right y-axis, percentage of genes; left y-axis, number of genes.

## Results and Discussion

### Short-read *De novo* Sequencing and Assembly

To obtain an overview of the *H. perforatum* gene expression profile over its entire growing cycle, cDNA samples from different developmental stages (vegetative stage, floral budding stage, and fresh fruiting stages) were prepared and RNA-seq was performed via Illumina HiSeq™ 2000. After trimming the adapter sequences and sequences that were less than 90 bases long, we obtained 24,429,306 clean paired-end reads with a total of 2,198,637,540 (2.2 GB) nucleotides. The Q20 percentage (sequencing error rate <1%) and GC percentage were 94.62% and 50.45%, respectively, and each read length was 90 bp×2. All reads were deposited in the NCBI and can be accessed in the Short Read Archive (SRA) under accession number SRA050246.2. We then applied SOAP2 *de novo* software [24] for assembling those short reads through a step-wise strategy. Eventually 59,184 unigenes (≥200 bp) were obtained, with an average length of 422 bp and an N50 of 532 bp (Table 1). Analysis of size distributions (Figure 1A) revealed that 69.47% fell within the range of 300 bp to 1,000 bp. Moreover, 98.98% unigenes showed no gap (Figure 1B).

### Functional Annotation and Gene Ontology Classification

To orient the unigenes derived from RNA-seq, we performed BlastX (version 2.2.21) alignment (e value <1.00E−05) against several protein databases: GenBank non-redundant (NR), Swiss-Prot, Clusters of Orthologous Groups (COG), Gene Ontology (GO), and Kyoto Encyclopedia of Genes and Genomes (KEGG). The best aligning results were used to decide the sequence direction of these unigenes. Here, 40,813 (68.86%) unigenes were oriented (Table 2).

COG (http://www.ncbi.nlm.nih.gov/COG/) is delineated by comparing protein sequences encoded in complete genomes and representing major phylogenetic lineages. Such an analysis provided us with function predictions and classifications. Some transcripts had multiple COG functions. Altogether, 11,207 unigenes were clustered into 24 functional categories (Figure 2A). Among them, the “General function prediction only” cluster was the largest (26.9%), followed by “Transcription” (16.11%). Another 698 unigenes (6.23%) belonged to the “Secondary metabolites biosynthesis” group. In that group, Cytochrome P450 had the most abundant sequences, with a total of 187 unigenes being involved in various pathways.

GO (http://www.geneontology.org/) is an international classification system for standardized gene functions, offering a controlled vocabulary and a strictly defined conceptualization for comprehensively describing the properties of genes and their products within any organism. The three main, independent GO categories are biological processes, molecular functions, and cellular components. With NR annotation, we used the Blast2GO (version 2.3.5) (http://www.blast2go.org/). Program [25] to obtain GO annotations and were able to map 15,145 (25.59%) unigenes to GO terms. Because a transcript sometimes had multiple terms, 15,145 of our unigenes could be summarized into the three main GO categories and then into 44 sub-categories. Of these, 10,562 (69.74%) comprised the largest category, molecular function, followed by cellular component (10,318, 68.13%) and biological process (8,721, 57.58%) (Figure 2B). Within the biological process group, the great majority was related to metabolic process (GO: 0008152, 70.85%) and cellular processes (GO: 0009987, 66.19%). Within cellular component, the largest proportion were assigned to cells (GO: 0005623, 98.73%), cell parts (GO: 0044464, 89.19%), and organelles (GO: 0043226, 70.01%). Approximately 70.85%, of those for cellular components pertained to catalytic activity (GO: 0003824), followed by binding (GO: 0005488, 63.42%).

**Figure 3:**
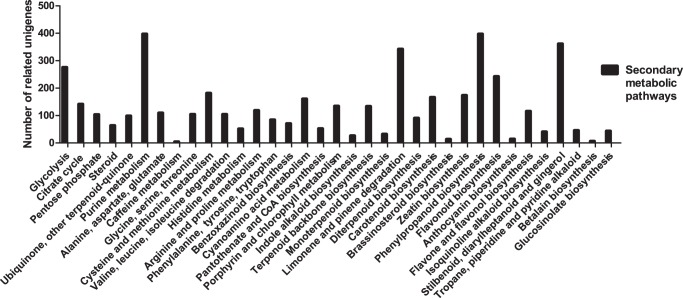
Unigenes from *Hypericum perforatum* related to secondary metabolic pathways.

Using the KEGG (http://www.genome.ad.jp/kegg/) database, we mapped 20,548 unigenes to 121 pathways. Among them, 4,148 were involved in metabolic pathways. Another 2,359, related to secondary metabolism, were mapped to 35 pathways (Figure 3). For example, the “Purine metabolism pathway” (ID: ko00230) and “phenylpropanoid biosynthesis pathway” (ID: ko00940) were the largest groups, containing 397 unigenes. The latter is the upstream pathway for flavonoid biosynthesis. We also found 242 and 115 unigenes in the “flavonoid biosynthesis pathway” (ID: ko00941) and “flavone and flavonol biosynthesis pathway” (ID: ko0094), respectively.

### Genes Related to Major Secondary Metabolism

**Figure 4:**
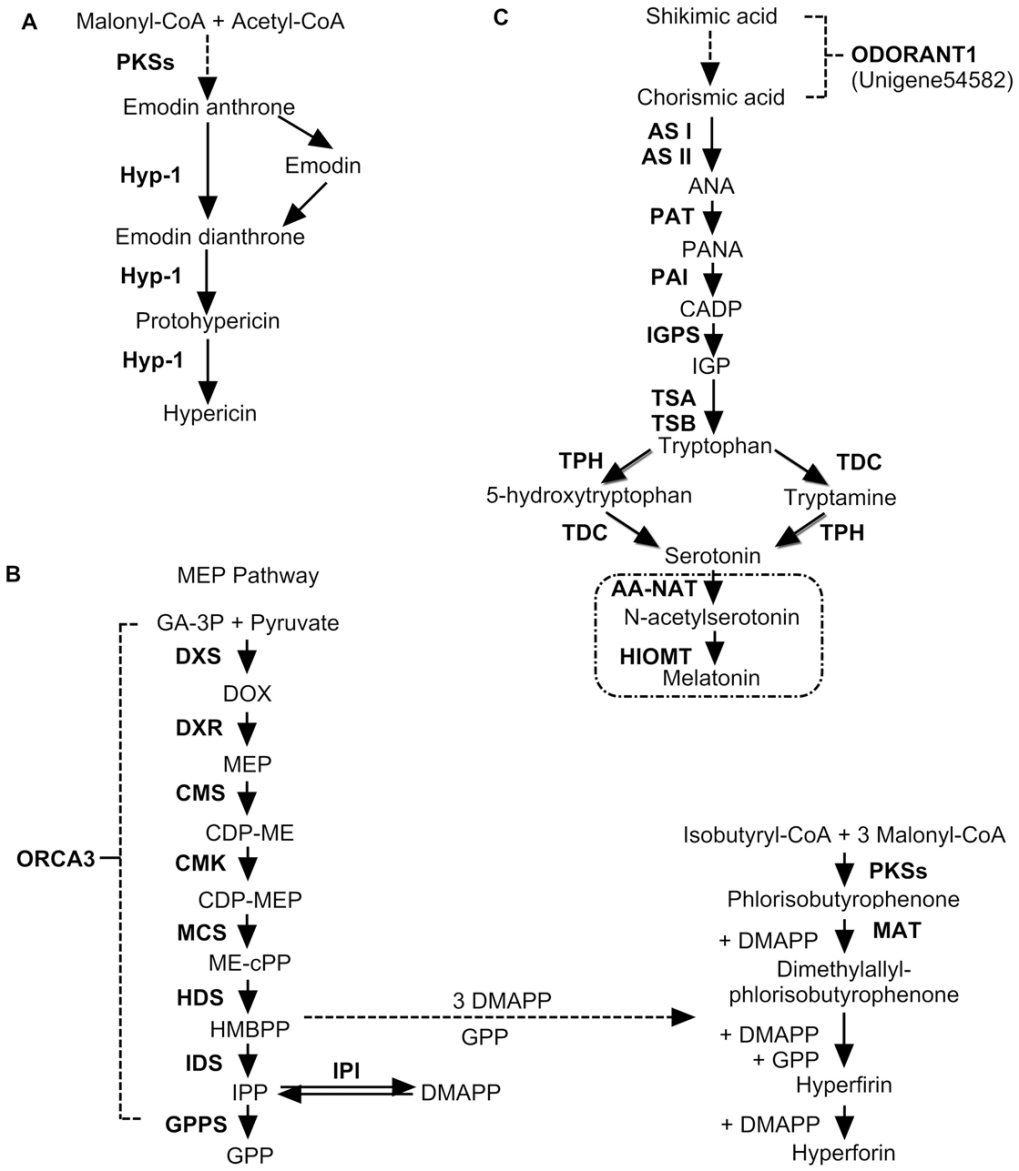
Putative biosynthesis pathways for hypericin (A), hyperforin (B), and melatonin (C) in *Hypericum perforatum*. Dashed box within (B) occurs in animals. Hyp-1, *Hypericum perforatum* phenolic oxidative coupling protein; MEP pathway, non-mevalonate pathway; MAT, dimethylallyltranstransferase; AS I, anthranilate synthase I; AS II, anthranilate synthase II; PAT, phosphoribosylanthranilate transferase; PAI, phosphoribosylanthranilate isomerase; IGPS, indole-3-glycerol phosphate synthase; TSA, Tryptophan synthase alpha chain; TSB, Tryptophan synthase beta chain; TDC, tryptophan decarboxylase; TPH, tryptophan hydroxylase; ORCA3, octadecanoid-derivative responsive *Catharanthus* AP2-domain protein 3; DXS, 1-D-deoxyxylulose 5-phosphate synthase; DXR, 1-deoxy-D-xylulose 5-phosphate reductoisomerase; CMS, 4-diphosphocytidyl-2-*C*-methyl-D-erythritol synthase; CMK, 4-(cytidine 5′-diphospho)-2-*C*-methyl-D-erythritol kinase; MCS, 2-C-methyl-D-erythritol 2,4-cyclodiphosphate synthase; HDS, (*E*)-4-hydroxy-3-methylbut-2-enyl-diphosphate synthase; IDS, isopentenyl-diphosphate:NAD(P)^+^ oxidoreductase; IPI, isopentenylpyrophosphate isomerase; GPPS, geranylgeranyl pyrophosphate synthase; DMAPP, dimethylallyl diphosphate; IPP, isopentenyl diphosphate; GPP, geranyl diphosphate; MEP, 2-C-methyl-Derythritol-4-phosphate; CDP-ME, 4-(cytidine-5′-diphospho)-2-C-methyl-Derythritol; CDP-MEP, 2-phospho-4-(cytidine-5′-diphospho)-2-C- methyl-Derythritol; Me-cPP, 2-C-methyl-D-erythritol-2,4, cyclodiphosphate; HMBPP, 1-hydroxy-2-methyl- 2-(E)-butenyl 4-diphosphate.

Biosynthesis of hypericin begins with the condensation of one molecule of acetyl-CoA with seven molecules of malonyl-CoA. The octaketide chain that forms subsequently undergoes cyclization and decarboxylation, leading to the formation of emodin anthrone (Figure 4A) [7]. For hypericin biosynthesis, Hyp-1 (*Hypericum perforatum* phenolic oxidative coupling protein) plays an important role in catalyzing that condensation reaction from emodin to hypericin [16]. Our annotated databases revealed 12 unigenes homologous to *Hyp-1* (Table 3).

**Table 3:** Putative unigenes related to the biosynthesis of hyperforin, hypericin, and melatonin.

		Enzyme	NU	MNCG
**Hyperforin and hypericin biosynthesis**	Type III PKSs	MAT	91	50
		Hyp-1	12	6
	MEP pathway	DXS	13	10
		DXR	2	2
		CMS	2	2
		CMK	2	1
		MCS	2	2
		HDS	4	1
		IDS	2	2
		IPI	2	2
		GPPS	62	49
**Melatonin biosynthesis**	Chorismate pathway	AS	12	7
		PAT	12	7
		PAI	2	2
		IGPS	2	1
		TSA	2	1
		TSB	8	5
	Tryptophan metabolism	TDC	11	11
		TPH	17	15
**Total**			260	126

NU, number of unigenes; MNCG, maximum number of coding genes. MAT, dimethylallyltranstransferase; Hyp-1, Hypericum perforatum phenolic oxidative coupling protein; MAT, dimethylallyltranstransferase; DXS, 1-D-deoxyxylulose 5-phosphate synthase; DXR, 1-deoxy-D-xylulose 5-phosphate reductoisomerase; CMS, 4-diphosphocytidyl-2-C-methyl-D-erythritol synthase; CMK, 4-(cytidine 5′-diphospho)-2-C-methyl-D- erythritol kinase; MCS, 2-C-methyl-D-erythritol 2,4-cyclodiphosphate synthase; HDS, (E)-4-hydroxy-3- methylbut-2-enyl-diphosphate synthase; IDS, isopentenyl-diphosphate:NAD(P)^+^ oxidoreductase; IPI, isopentenylpyrophosphate isomerase; GPPS, geranylgeranyl pyrophosphate synthase; AS, anthranilate synthase; PAT, phosphoribosylanthranilate transferase; PAI, phosphoribosylanthranilate isomerase; IGPS, indole-3-glycerol phosphate synthase; TSA, Tryptophan synthase alpha chain; TSB, Tryptophan synthase beta chain; TDC, tryptophan decarboxylase; TPH, tryptophan hydroxylase.

The biosynthesis of hyperforins can be divided into two phases of formation – carbon skeletons and prenyl side chains (Figure 4B) [8], [26], [27]. This skeleton starts from one molecule of isobutyryl-CoA and three molecules of malonyl-CoA that undergo a condensation reaction catalyzed by type III PKS (known as isobutyrophenone synthase, or BUS). Prenylation of that skeleton accepts the prenyl from an isoprenoid, which is biosynthesized via the non-mevalonate pathway (MEP pathway) [25]. Three molecules of dimethylallyl pyrophosphate and one molecule of geranyl diphosphate join in modifying those prenyl side chains to yield hyperforin. We identified more than 91 unigenes from our database and determined that they are involved in the entire MEP pathway. This is the first time all of these genes have been identified in *H. perforatum* (Table 3). We also identified 91 unigenes homologous to dimethylallyltranstransferase (MAT) gene from our database.

Although tryptophan biosynthesis has been clearly described in *Arabidopsis* [28], the pathway from tryptophan to melatonin is still unclear. In mammals, yeast, and bacteria, melatonin is synthesized from tryptophan via 5-hydroxytryptophan, tryptamine, and serotonin [29]. In *H. perforatum*, melatonin is synthesized from tryptophan via 5-hydroxytryptophan and serotonin [15]. We drew a putative melatonin biosynthetic pathway for that species as well (Figure 4C). In our database, we found 66 unigenes encoding nine enzymes involved in melatonin biosynthesis, including anthranilate synthase (AS) I and II, phosphoribosylanthranilate transferase (PAT), phosphoribosylanthranilate isomerase (PAI), indole-3-glycerol phosphate synthase (IGPS), tryptophan synthase (TSA and TSB), tryptophan decarboxylase (TDC), and tryptophan hydroxylase (TPH) (Table 3). This is first time that any of these have been identified in *H. perforatum*.

### Prediction of Type III Polyketide Synthase

Type III PKS is a class of enzymes that catalyzes the synthesis of polyketides, such as CHS, BUS, and STS. In higher plants, the CHS-type shows >80% similarity with chalcone synthases and >70% similarity with non-chalcone synthases, or STS- and CTAS-types [30].

Only five type III PKS proteins – benzophenone synthase, octaketide synthase, chalcone synthase, HyPKS1, and HyPKS2– have previously been reported from *H. perforatum* [17]. Here, we used PKSIIIexplorer (http://type3pks.in/tsvm/pks3/index.php) to predict unigenes encoding such proteins [31], and obtained 2,291 (3.87%) unigenes (Table S1). In that species, polyketides may have dual functions during biotic stress: 1) as antioxidants to protect cells from oxidative damage, and 2) as phytoalexins to inhibit the growth of pathogens [32], [33]. Our results provide an overview of type III PKSs in *H. perforatum* that will facilitate further studies.

### Identification and Analysis of Transcription Factors

Transcription factors (TFs) affect metabolic flux by regulating related encoding enzymes gene expression. Their identification offers information for manipulating secondary metabolism in medicinal plants. Here, BlastX was performed to search against the AGRIS (*Arabidopsis* Gene Regulatory Information Server) database [34]. In all, 1,772 unigenes were annotated in 744 independent coding sequences of *Arabidopsis* TFs (identity >77%) that belong to 47 known TF families (Table 4). For example, 212, 189, 144, 132, 120, 106, and 95 unigenes were annotated to NAC, C2H2, AP2-EREBP, C3H, Homeobox, bHLH, and MYB families, respectively.

**Table 4:** Putative transcription factors encoding unigenes in *Hypericum perforatum*.

TF Family	NU	NATHB	NTGAD	Percentage(%)
NAC	212	43	96	44.79
C2H2	189	89	211	42.18
AP2-EREBP	144	53	138	38.40
C3H	132	52	165	31.52
Homeobox	120	55	102	53.92
bHLH	106	56	161	34.78
MYB	95	51	208	24.52
MADS	86	27	111	24.32
WRKY	77	34	72	47.22
bZIP	52	31	73	42.47
GRAS	52	15	33	45.45
G2-like	43	18	40	45.00
Trihelix	39	19	29	65.52
ARF	33	11	24	45.83
Other	392	190	452	42.04

NU, number of unigenes; NATHB, number of *Arabidopsis* TF genes hit by Blast; NTGAD, number of TF genes in AGRIS database; percentage  =  NATHB/NTGAD.

AP2/EREBP, bHLH, and MYB are important TF families regulating secondary metabolism in plants, playing an important role in the control of indole alkaloid and tryptophan biosynthesis [35]–[37]. The octadecanoid-derivative responsive *Catharanthus* AP2-domain protein 3 (ORCA3) activates the expression of several genes that encode enzymes involved in indole alkaloid biosynthesis and MEP pathway, e.g., AS I, TDC, and 1-D-deoxyxylulose 5-phosphate synthase (DXS). Altered tryptophan regulation 1 (ATR1) – a MYB factor – and altered tryptophan regulation 2 (ATR2) – a bHLH factor – activate genes that function in tryptophan biosynthesis and metabolism in *Arabidopsis* [36], [37].

### Real-time PCR Analysis of Several Novel Transcripts

**Figure 5:**
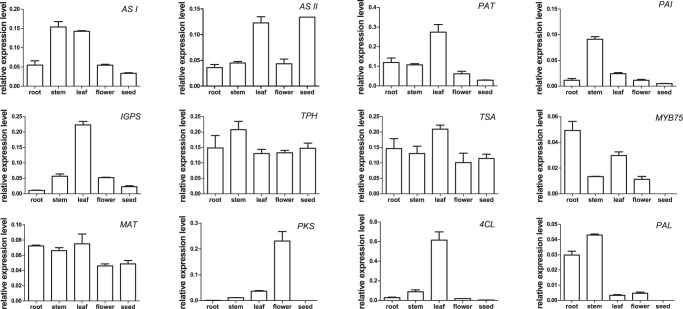
Expression patterns of some novel transcripts related to secondary metabolism in *Hypericum perforatum*. ERROR BARs indicate standard deviation.

For a better understanding of metabolites, one must also evaluate the temporal and spatial expression profiles of key genes. Our BlastX alignment produced the best aligning results for *HyAS I*, *HyAS II*, and *HyPAT*. We then performed RT-PCR analysis to investigate the expression patterns of 12 novel transcripts (Figure 5; Table S2). Within the melatonin biosynthesis pathway, *AS I*, *PAI*, and *TPH* were highly expressed in the stems, whereas *PAT*, *IGPS*, and *TSA* were mainly expressed in the leaves. In addition, *AS II* was highly expressed in the leaves and seeds. It is generally accepted that tryptophan is biosynthesized in the chloroplasts [38], [39]. Our results are consistent with previous findings that the genes involved in tryptophan biosynthesis have high expression in the leaves and stems, both containing chloroplasts. We noted that the unigene44757 homolog of *GmMYB75*, an R2R3-MYB family member, was highly expressed in roots but very little in the seeds. To better known the function and expression pattern of unigene44757, further researches are needed.

In the hyperforin biosynthesis pathway, *MAT* was expressed in all tissues, albeit at slightly higher levels in the leaves. *PKS* was mainly expressed in flowers but only minimally in the roots and seeds. These results support previous findings that polyketide is more abundant at the flowering stage. *4CL* was expressed mainly in the leaves while *PAL* was highly expressed in the stems and roots. These two genes involved in the phenylpropanoid pathway showed different patterns that were not consistent with those of genes in other species [40], [41].

### SSR Frequency and Distribution

Simple Sequence Repeats (SSRs) or microsatellites are ubiquitous in eukaryotic genomes. They are distributed in both coding and non-coding regions [42]. Their varied lengths affect the expression patterns of certain genes. SSRs are ideal for determining paternity, investigating population genetics, and recombination mapping, and they are considered the only molecular marker for providing clues about which alleles are more closely related [43].

In all, we identified 7,643 SSRs as potential molecular markers for genetics applications. Dinucleotide repeats (3,019) were the most common SSRs in our datasets. This distribution is consistent with that in most other dicotyledonous species, such as *Arabidopsis*, peanut, and grape [44]. The second major class was trinucleotide (1,990), followed by mononucleotide (1,317), hexanucleotide (586), pentanucleotide (482), and tetranucleotide (249) (Table 5). Di- and trinucleotides frequently showed five repeats while penta- and hexa- had two. The mononucleotide often appeared in 10 to 14 repeats. SSRs with five tandem repeats (30.37%) were the most common.

**Table 5:** Number of SSRs in *Hypericum perforatum*.

Repeat motif	Repeat numbers								Number of SSRs	Percent (%)
	3	4	5	6	7	8	9	>9		
Mono-	–	–	–	–	–	–	–	1317	1317	17.23
Di-	–	–	1101	405	245	216	200	852	3019	39.5
Tri-	–	–	1117	407	198	83	50	135	1990	26.04
Tetra-	–	181	49	13	5	1	0	0	249	3.26
Penta-	377	76	23	3	1	2	0	0	482	6.31
Hexa-	453	89	31	13	0	0	0	0	586	7.67
Total	830	346	2321	841	449	302	250	987	7643	
Percent(%)	10.86	4.53	30.37	11	5.87	3.95	3.27	12.91		

Table 6 presents the frequencies of these di- and trinucleotide repeats. In our database, the AG/CT motif was the dominant repeat motif (up to 33.95%), followed by AAG/CTT (8.18%); CG/GC (0.2%) occurred very infrequently. The results are consistent with those reported from other plant species [44]–[46].

**Table 6:** Frequency of di- and trinucleotide EST-SSR repeat motifs in *Hypericum perforatum*.

Repeat motif	Repeat numbers						Total	Percent(%)
	5	6	7	8	9	>9		
AC/GT	147	42	11	13	12	17	242	3.17
AG/CT	815	338	223	199	186	834	2595	33.95
AT/AT	127	22	11	4	2	–	166	2.17
CG/GC	12	3	–	–	–	1	16	0.2
AAC/GTT	83	32	17	8	1	7	148	1.94
AAG/CTT	289	122	60	34	22	98	625	8.18
AAT/ATT	48	17	12	1	2	1	81	1.06
ACC/GGT	80	34	14	2	–	–	130	1.7
ACG/CGT	54	17	3	1	1	1	77	1.01
ACT/AGT	17	4	–	1	1	1	24	0.31
AGC/CTG	76	25	10	2	1	1	115	1.5
AGG/CCT	320	123	61	25	13	2	554	7.25
ATC/ATG	83	26	18	9	9	14	159	2.08
CCG/CGG	67	7	3	–	–	–	77	1.01

## Conclusions

Because *Hypericum perforatum* is the main natural source for extracted hypericin and hyperforin, research of this plant is on-going. Its pharmacological properties are gradually being revealed, including those that are anti-tumor, anti-inflammatory, antiviral, anti-cancer, and antibacterial. Likewise, some health-promoting compounds, such as melatonin, are products of secondary metabolism. Our study is the first to use Illumina/Solexa deep sequencing for identifying 59,184 unigenes within the *H. perforatum* gene pool. This enriched genetic information not only provides us with an insight into the molecular mechanisms of various metabolic pathways, but also enables us to improve our efforts in genetic manipulations and characterize species specific genes. This is an important public information platform for better understanding gene expression, genomics, and functional genomics in this valuable species.

## Materials and Methods

### Ethics Statement

No specific permits were required for the described field studies. No specific permissions were required for these locations and activities. The location is not privately-owned or protected in any way and the field studies did not involve endangered or protected species.

### Plant Material

Seeds of *Hypericum perforatum* L. (Hypericaceae) were harvested from wild populations at Taibai Mountain (Shaanxi Province, China), and were identified by Dr. Ren Yi (Shaanxi Normal University). Plant voucher specimens (TB-Hp-001) have been conserved in the herbarium of College of Life Sciences at Shaanxi Normal University. The seeds were germinated in pots containing vermiculite. Conditions in the climate cabinet included a temperature of 25°C and a 16-h photoperiod (150 µmol·m^−2^·s^−1^). Plant materials available at each time point (i.e., roots, stems, leaves, and flowers) were collected at the vegetative stage (I, two-month-old seedlings), floral budding stage (II), and fresh fruiting stages (III). All samples were frozen immediately in liquid nitrogen and stored at −80°C.

### RNA Isolation and Sequencing

Total RNAs were extracted with an Omega Plant RNA Kit (with DNase I) according to the manufacturer’s instructions. A single RNA sample was pooled from Stages I, II, and III. An RNase inhibitor (RNAlong; BioTeke, Beijing, China) was added to the RNA samples before they were analyzed by BGI (Shenzhen, Guangdong, China). Afterward, a cDNA library was constructed and RNA-seq was performed via Illumina HiSeq™ 2000.

Total RNAs from various organs at Stage II (roots, stems, leaves, and flowers) were extracted separately, using an Omega Plant RNA Kit with DNase I. We also extracted total RNAs from the seeds, as described above. Single-stranded cDNAs for real-time PCR analysis were synthesized from RNAs, using a PrimeScriptTM 1st Strand cDNA Synthesis Kit (TaKaRa, Dalian, China).

### *De novo* Transcriptome Assembly and Annotation

We obtained 25,666,478 raw reads. After removing those with only adaptors, with unknown nucleotides larger than 5%, or those that were of low quality, only clear reads remained. These were then assembled for unigene annotation so that we could classify them for gene functioning as we have previously described [24].

### Prediction of Type III PKSs

PKSIIIexplorer is a web server based on the “Transductive Support Vector Machine” that allows for fast and reliable predictions of type III PKS proteins [9], [31]. As candidates, we used peptide sequences predicted from 40,813 unigenes in our blast results. Putative type III PKS or type III PKS -like proteins received positive scores; all others were scored negatively.

### RT-PCR and Expression Analysis

RT-PCR analysis was used to evaluate the quality of the sequence assembly. Twelve transcripts were chosen for monitoring their expression patterns (Table S2). We utilized the IQ5 real-time PCR detection system (Bio-Rad) as we have previously described [24]. Glyceraldehyde-3-phosphate dehydrogenase (GAPDH, GU014528) served as an internal reference gene, and relative expression was calculated per the 2^−ΔΔCt^ method [47]. All quantitative PCR runs were repeated in three biological and three technical replications.

### SSR Analysis and TF Identification

SSRs were detected among the 59,184 unigenes via MIcroSAtellite (MISA, http://pgrc.ipk-gatersleben.de/misa/). The parameters were adjusted in order to identify perfect mono-, di-, tri-, tetra-, penta-, and hexanucleotide motifs with a minimum of 10, 5, 5, 4, 3, and 3 repeats, respectively. A Blast search for all unigenes was conducted with AtTFDB (*Arabidopsis* transcription factor database) to find TFs with shared identities >77%.

## Supporting Information

Table S1 **Putative Type III PKSs in** ***Hypericum perforatum*** **.**
(XLS)

Table S2 **Gene-specific primers used for gene expression analysis by quantitative real-time PCR.**
(DOC)
