# Supplementary material for: De Novo Sequencing of Hypericum perforatum Transcriptome to Identify Potential Genes Involved in the Biosynthesis of Active Metabolites
Source: PLoS One. 2012 Jul 30;7(7):e42081. doi: 10.1371/journal.pone.0042081 (PMC3408400; doi:10.1371/journal.pone.0042081)
Supplement: Table S2 — Gene-specific primers used for gene expression analysis by quantitative real-time PCR. (DOC) [file pone.0042081.s002.doc]

**Table S**2 Gene-specific primers used for gene expression analysis by quantitative real-time PCR.

| **Gene** | **Unigene** | **Primers (5’-3’)** |
| --- | --- | --- |
| ***AS I*** | unigene2994 | TTG AGT CTG TTG AGC CTG GTT TT (forward) |
|  |  | ACT GCG ACC TTA TTT TCT CTT GCT (reverse) |
| ***AS II*** | unigene61620 | GCA TTG GAG AGG CTT TTG G (forward) |
|  |  | GAC CAT CTT CCC CTT TCT CAT C (reverse) |
| ***PAT*** | unigene80084 | ACC AGG GTT GGC TTT GAC C (forward) |
|  |  | TTC TTC CTC CGT CCT CTC TCC (reverse) |
| ***PAI*** | unigene66472 | GCA GGT TTG AGG ATA GGA AGA TTG (forward) |
|  |  | GTT CTC GGG TGA AGC ATA AAT ACA C (reverse) |
| ***IGPS*** | unigene77522 | AAC GGG GCC GGA TAA TTC (forward) |
|  |  | CAC CAA AAC CGC TCT CAC AC (reverse) |
| ***TPH*** | unigene6952 | TCC GAC ACC GCT TCA ACC T (forward) |
|  |  | GGG AAC GGG AGA AGG AAA AC (reverse) |
| ***TSA*** | unigene76727 | GGA CTT GTG GTG CCT GAT GTT (forward) |
|  |  | TTC TTC TGG GGT GGT AGG TGT T (reverse) |
| ***MYB75*** | unigene44797 | GAG GTT TCT GAT GGG GCT AAT G (forward) |
|  |  | GCT GGC TAC TTG GGT TGG AG (reverse) |
| ***MAT*** | unigene79971 | GAA CAT GGT TTG AGA ACC GAC A (forward) |
|  |  | ATA CAC AGA CGA TAG AGC GAC GAG (reverse) |
| ***PKS*** | unigene3482 | CGG GAA TGT GAG CAG CAA (forward) |
|  |  | CCA GGA CCA AAA GCC AAA G (reverse) |
| ***4CL*** | unigene12219 | CGA GAA GAT GTG GAA CAG AGG A (forward) |
|  |  | AGC AGG TGG ACG GAG AAA AC (reverse) |
| ***PAL*** | unigene57532 | TGT GAG GCA ACG TGT GAA TAG A (forward) |
|  |  | TCC CAT AGA AGG ACC AAG CAA (reverse) |
| ***GAPDH*** | GU014528.1 | ATG GAC CAT CAA GCA AGG ACT G (forward) |
|  |  | GAA GGC CAT TCC AGT CAA CTT C (reverse) |
